# An Environmentally-Friendly Three-Dimensional Computer-Aided Verification Technique for Plastic Parts

**DOI:** 10.3390/polym14152996

**Published:** 2022-07-24

**Authors:** Chil-Chyuan Kuo, Zong-Yan He, Chil-Xian Lee

**Affiliations:** 1 Department of Mechanical Engineering, Ming Chi University of Technology, No. 84, Gungjuan Road, New Taipei City 243, Taiwan; u08117205@mail.mcut.edu.tw; 2 Research Center for Intelligent Medical Devices, Ming Chi University of Technology, No. 84, Gungjuan Road, New Taipei City 243, Taiwan; 3 Road Ahead Technologies Consultant Corporation 8F, No. 88-11, Sec. 1, Guangfu Rd., Sanchong Dist., New Taipei City 241, Taiwan; ricklee@gmail.com

**Keywords:** plastic component, sustainable development goals, green three-dimensional optical inspection technology, shrinkage, non-contact computer-aided verification

## Abstract

Plastic components play a significant role in conserving and saving energy. Plastic products provide some advantages over metal, including reducing part weight, manufacturing costs, and waste, and increasing corrosion resistance. Environmental sustainability is one of the sustainable development goals (SDGs). Currently, the non-contact computer-aided verification method is frequently employed in the plastic industry due to its high measurement efficiency compared with the conventional contact measuring method. In this study, we proposed an innovative, green three-dimensional (3D) optical inspection technology, which can perform precise 3D optical inspection without spraying anything on the component surface. We carried out the feasibility experiments using two plastic parts with complex geometric shapes under eight different proposed measurement strategies that can be adjusted according to the software interface. We studied and analyzed the differences in 3D optical inspection for building an empirical technical database. Our aim in this study is to propose a technical database for 3D optical measurements of an object without spraying anything to the component’s surface. We found that the research results fulfilled the requirements of the SDGs. Our research results have industrial applicability and practical value because the dimensional average error of the two plastic parts has been controlled at approximately 3 µm and 4.7 µm.

## 1. Introduction

Plastic parts [1,2,3,4,5] can meet a new product’s useful requirements compared with metal parts. A new model vehicle is low in weight compared with old vehicles because its metal parts were replaced by plastic parts. The non-contact measuring method [6] is widely used in current polymer engineering because of its high measurement efficiency compared with the conventional contact measuring method [7]. Wang et al. [8] developed the structured-light 3D scanner for the quality assurance of a Ti-6Al-4V part fabricated by the additive manufacturing technique [9,10,11]. The proposed scanner improved the scanning and processing speed from 2 to 20 s compared with the conventional white light interferometer. Rękas et al. [12] proposed an approach to check the geometry of stamped car body parts using an optical 3D scanner. Research results revealed that this approach greatly accelerates the error correction process. Affatato et al. [13] assessed the wear of mobile total knee polyethylene inserts using an optical 3D scanner and investigated its wear behavior. Valigi et al. [14] demonstrated an innovative method to detect and evaluate the wear of biomedical devices and industrial components. Affatato et al. [15] detected the wear distribution of knee joint prostheses using an optical 3D scanner. It was found that the mobile total knee prosthesis has a lower wear resistance.

**Figure 1:**
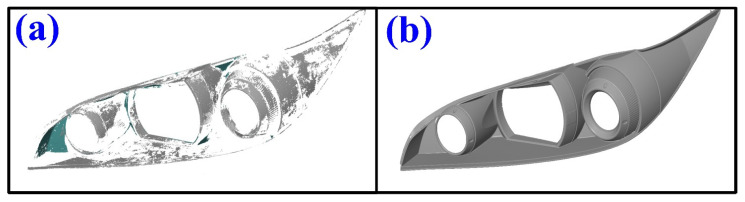
Measurement results of measurement object (**a**) without and (**b**) with spraying mixture of TiO_2_ powder and ethanol.

Currently, a non-contact GOM ATOS Triple Scan II optical 3D scanner is frequently employed in various engineering processes because it utilizes structured blue light to perform precise scans with detailed resolutions at high speeds. Currently, sensors are designed as flexible 3D scanners for complicated inspection tasks in various industries. In practical engineering, the conventional method is that the measurement object is matted by spraying an anti-glare mixture of titanium oxide (TiO_2_) powder and ethanol for achieving optimal accuracy and results in the optical 3D measurement. Figure 1 shows the measurement results of the measurement object with and without spraying a mixture of TiO_2_ powder and ethanol. However, three major disadvantages of spraying an anti-glare mixture of TiO_2_ powder and ethanol on the measured object were found. Firstly, the time to spray a mixture of TiO_2_ powder and ethanol will be longer when the size of the measurement object is larger. In addition, the cost of the pre-work will be increased. Secondly, removing the spraying mixture is time-consuming when the measurement object has many fine pores or seams. Finally, the measurement object could be damaged while removing the spraying mixture after the 3D optical measurement.

However, some customers asked for the measurement object to be scanned directly without spraying a mixture of TiO_2_ powder and ethanol. Four distinct advantages of not spraying an anti-glare mixture of TiO_2_ powder and ethanol on the measured object include no damage to the measurement object, saving lead time before 3D optical measurements, and cost savings. Therefore, the main objective of this work is to propose an innovative, green 3D computer-aided verification (CAV) technique using an ATOS optical 3D scanner, which can perform precise 3D optical inspections of a measured object without spraying a mixture of TiO_2_ powder and ethanol. The feasibility experiments were performed using eight different measurement methods and two different measurement objects. The differences in 3D optical inspection between samples with and without the mixture of TiO_2_ powder and ethanol were experimentally investigated. Finally, an empirical technical database was built.

## 2. Experimental Details

**Figure 2:**
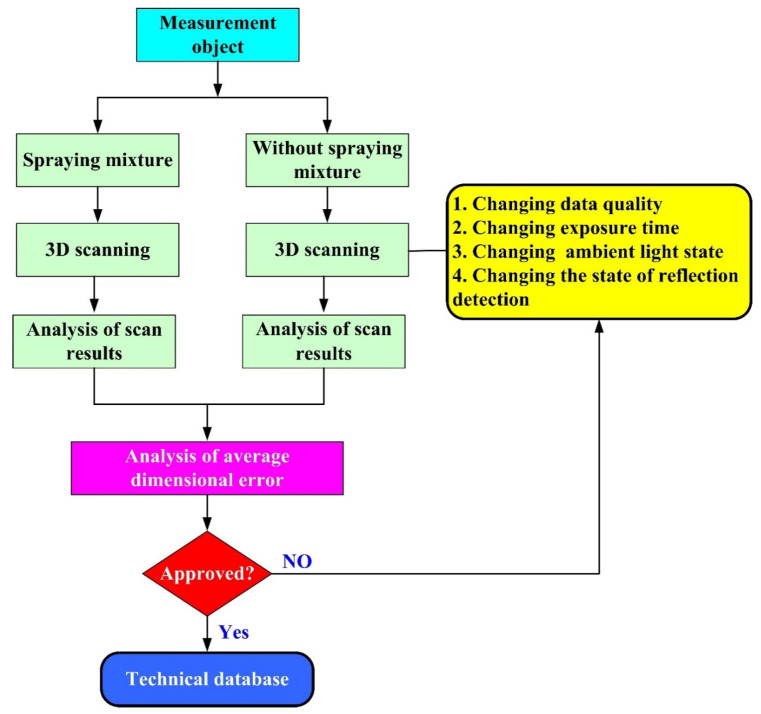
Flow diagram of experimental methodology.

**Figure 3:**
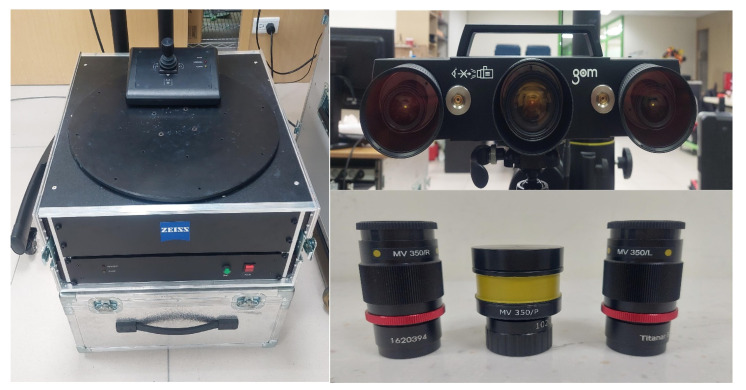
3D digitizing instruments used in study.

**Figure 4:**
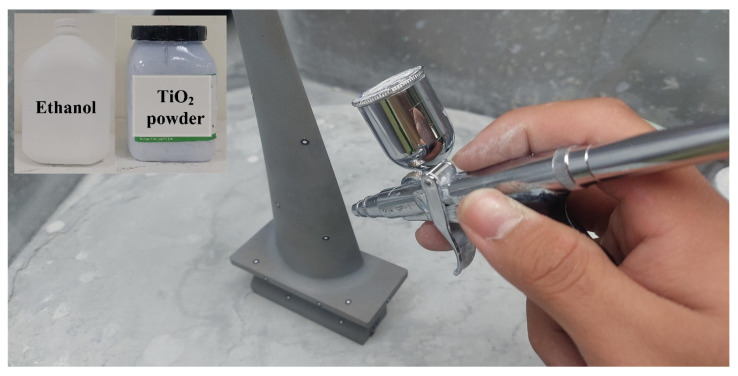
Situation of object sprayed with mixture of TiO_2_ powder and ethanol.

Figure 2 shows the research process of this study. In practical engineering, calibration of an ATOS sensor using a calibration frame before optical measurement is required. We sprayed the surfaces of the measurement objects to avoid undesired reflections during optical measurement. Figure 3 shows the 3D digitizing instruments used in this study, namely GOM ATOS Triple Scan II optical 3D scanner (ATOS, GOM Inc., Essen, Germany). We used a 3D optical scanner with blue light source to quantify the dimensions of the measurement objects [12,16,17]. We mixed the TiO_2_ powder [13,18] with 95% ethanol in a weight ratio of 1:4 to create the mixture. The average particle size of TiO_2_ powder is approximately 4 µm. The main reason we selected TiO_2_ powder as the main compound of the mixture is that TiO_2_ powder has a high refractive index of about 2.87. Thus, it is easy to form total reflection when the measurement object is sprayed with a mixture of TiO_2_ powder and ethanol during 3D optical measurement. In this study, we employed two lenses (MV350 and MV500) and one rotation stage (ROT 640) for 3D optical measurements. The GOM 3D software adjusts the scan data quality, resolution, area, and exposure. Our main purpose for sticking reference points on the surfaces of the measurement objects was to make the scanning probe remember the relative position of the measurement object. Thus, the scanned data can be superimposed by the number of the reference points. We projected the stripe patterns on the surfaces of measurement objects and captured images with two cameras. We obtained results by applying GOM Software and fundamental theoretical concepts concerning the sensor. Finally, we determined the 3D coordinate measuring data from the beam paths of both cameras and projector. Figure 4 shows the situation of the object sprayed with mixture of TiO_2_ powder and ethanol. The spraying process parameters involve a spraying distance of 10 cm, an angle of about 15°, and a mixture speed of about 14.4 cm/s.

In this study, data measured by spraying a mixture of TiO_2_ powder and ethanol were selected as the control group. The data from eight different measurement methods were used as the eight test groups. According to the software interface, eight different measurement strategies can be adjusted. Table 1 shows the number of test groups and measurement strategies. We tilted the sensor head at an angle of about 15° to 20° to prevent it from directly illuminating the measuring object and generating unnecessary reflected light. In experiment 1, we investigated the differences between high quality and more points in the 3D optical measurement results for an object that was not sprayed with a mixture of TiO_2_ powder and ethanol. In experiment 2, we investigated the differences between one and two exposure times in the 3D optical measurement results for an object that was not sprayed with a mixture of TiO_2_ powder and ethanol. In experiment 3, we investigated the effects of turning on the reflection detection on the 3D optical measurement results for an object that was not sprayed with a mixture of TiO_2_ powder and ethanol. In experiment 4, we investigated the effects of turning on reflection detection under two exposure times on the 3D optical measurement results for an object that was not sprayed with a mixture of TiO_2_ powder and ethanol. In experiment 5, we investigated the effects of turning off ambient light on the 3D optical measurement results for an object that was not sprayed with a mixture of TiO_2_ powder and ethanol. In experiment 6, we investigated the effects of turning off ambient light under two exposure times on the 3D optical measurement results for an object that was not sprayed with a mixture of TiO_2_ powder and ethanol. In experiment 7, we investigated the effects of reflection detection and turning off ambient light in the 3D optical measurement results for an object that was not sprayed with a mixture of TiO_2_ powder and ethanol. In experiment 8, we investigated the effects of reflection detection and turning off ambient light under two exposure times on the 3D optical measurement results for an object that was not sprayed with a mixture of TiO_2_ powder and ethanol.

According to practical experience in the industry, it is not easy to perform 3D optical inspection on transparent plastic objects because the surface of the measurement object is not reflective. The industry is divided into two categories according to the appearance color of plastic-injection-molded parts. One is the light-colored plastic part. The other is the dark plastic part. Thus, a precision-injection-molding mold can be implemented using the assistance of CAV technique. Table 2 shows the number of the measurement objects and their characteristics. The characteristics of measurement object 1 include reflections, but the brightness and area are less than those of dark plastic parts. The characteristics of measurement object 2 include reflections, but the brightness and area are more than light-colored plastic parts.

## 3. Results and Discussion

**Figure 5:**
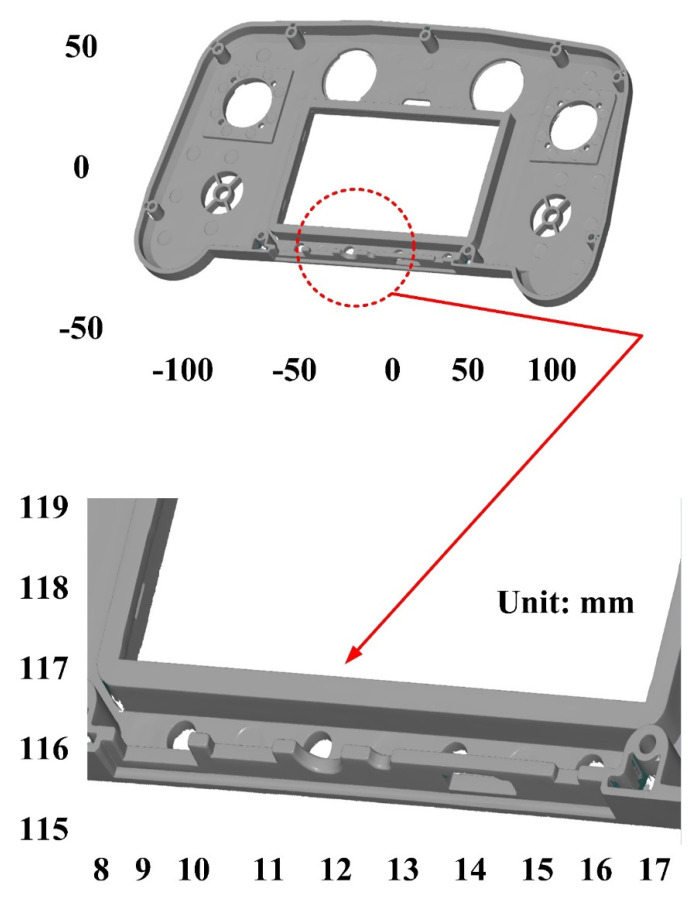
Measurement results of light-colored plastic part sprayed with mixture of TiO_2_ powder and ethanol.

**Figure 6:**
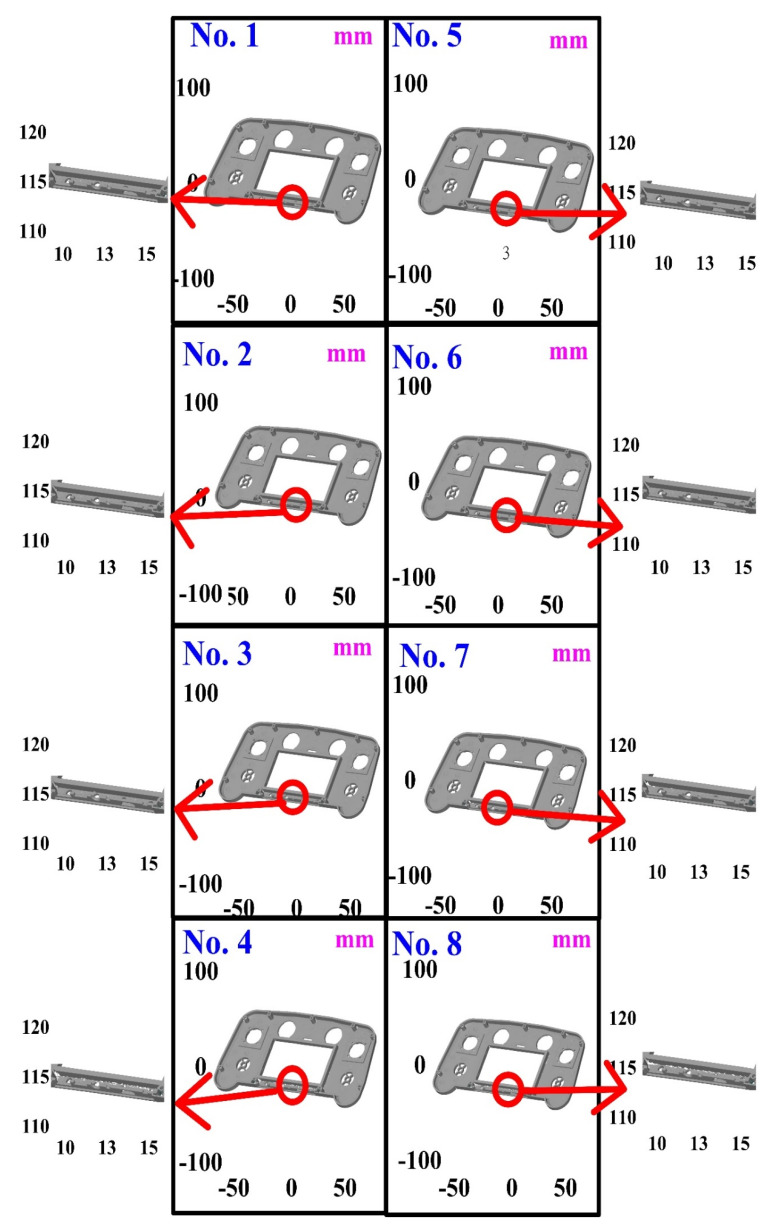
Measurement results of light-colored plastic part without sprayed mixture of TiO_2_ powder and ethanol.

**Figure 7:**
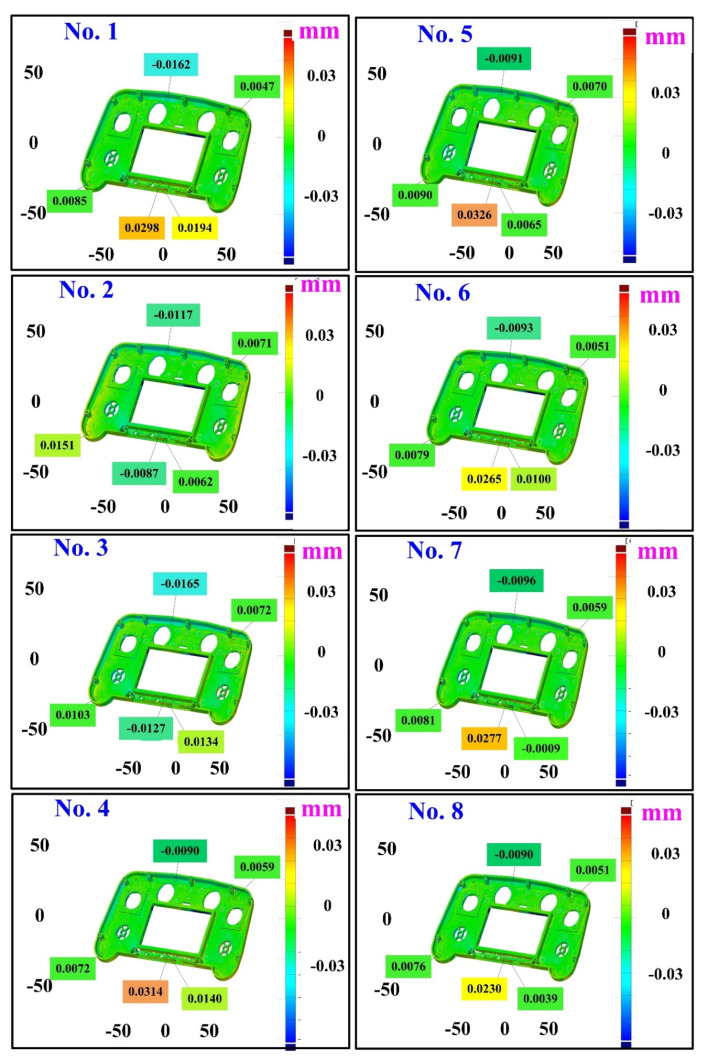
CAV results of light-colored plastic part. Data for five measurements in figure are only representative analysis results.

**Figure 8:**
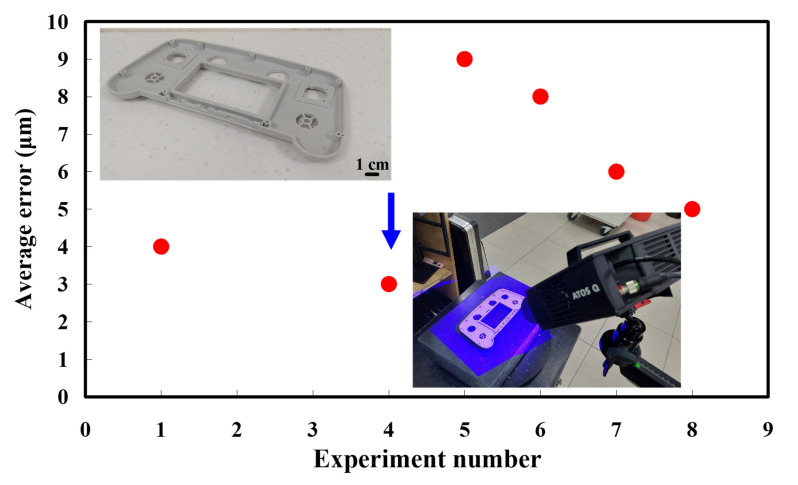
Average error of eight different measurement methods compared with conventional method for light-colored plastic part.

The first measurement object is a touch panel, which is a light-colored plastic part. This part is made of polycarbonate (PC)/acrylonitrile butadiene styrene (ABS) [19,20,21]. This plastic body does not produce a lot of reflections. However, the surfaces at the corners of the plastic parts are relatively smooth, and are prone to reflecting light. Figure 5 shows the measurement results of the light-colored plastic part sprayed with a mixture of TiO_2_ powder and ethanol. The number of measurement points using high quality functions is about 347,460. Figure 6 shows the measurement results of the light-colored plastic part that was not sprayed with a mixture of TiO_2_ powder and ethanol. The number of measurement points for the eight different measurement methods is approximately 506,867, 490,685, 536,381, 499,777, 549,488, 529,030, 548,772, and 496,654, respectively. As can be seen, the eight 3D digital models were reconstructed by the structured-light 3D scanner using eight different measurement methods. The control group is considered as the nominal model and the color deviation map stands for the measurement results deviated from the nominal model. Figure 7 shows the CAV results of the light-colored plastic part. It should be noted that the data of the five measurements in the figure are only representative analysis results. Figure 8 shows the average error of the eight different measurement methods compared with the conventional method for the light-colored plastic part. The average size errors of the eight different measurement methods are approximately 4 µm, 6 µm, 6 µm, 3 µm, 9 µm, 8 µm, 6 µm, and 5 µm, respectively, compared with the overall measurement results of the control group. In particular, the average size error of the 3D optical measurement parameters using experiment 4 is the smallest. The average size error is only 3 µm. Therefore, the optimal measurement parameters for the light-colored plastic part sprayed with a mixture of TiO_2_ powder and ethanol are recommended to be full resolution, more point, reflection detection on, ambient light on, and two exposure times.

**Figure 9:**
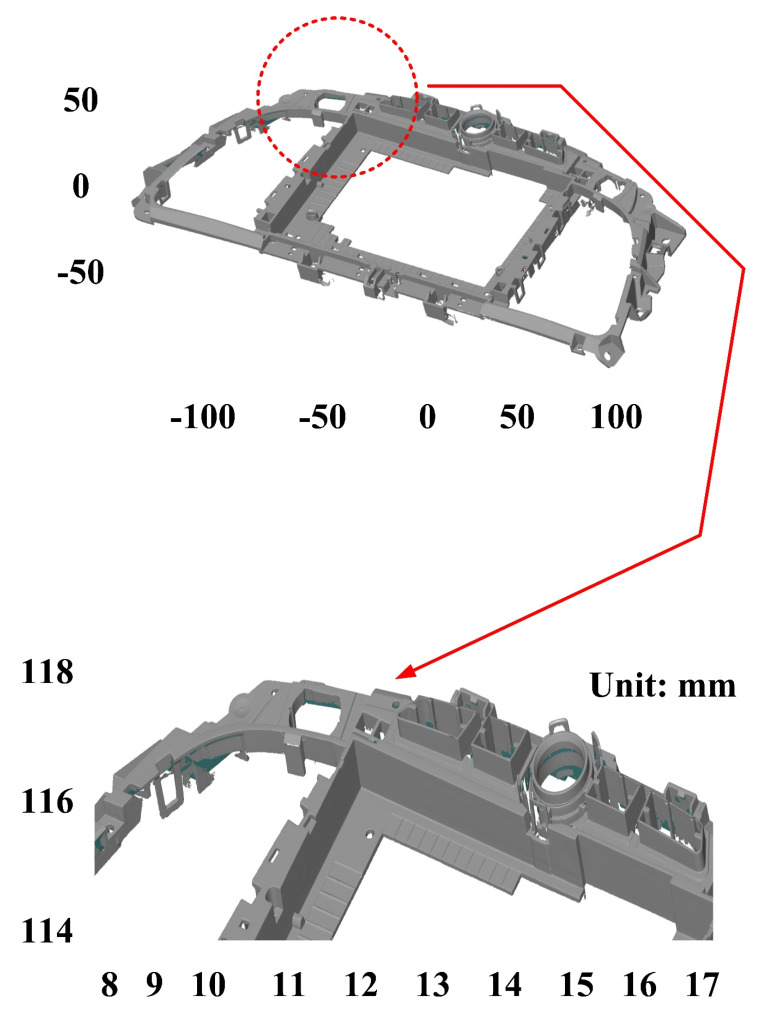
Measurement results of dark plastic part sprayed with mixture of TiO_2_ powder and ethanol.

**Figure 10:**
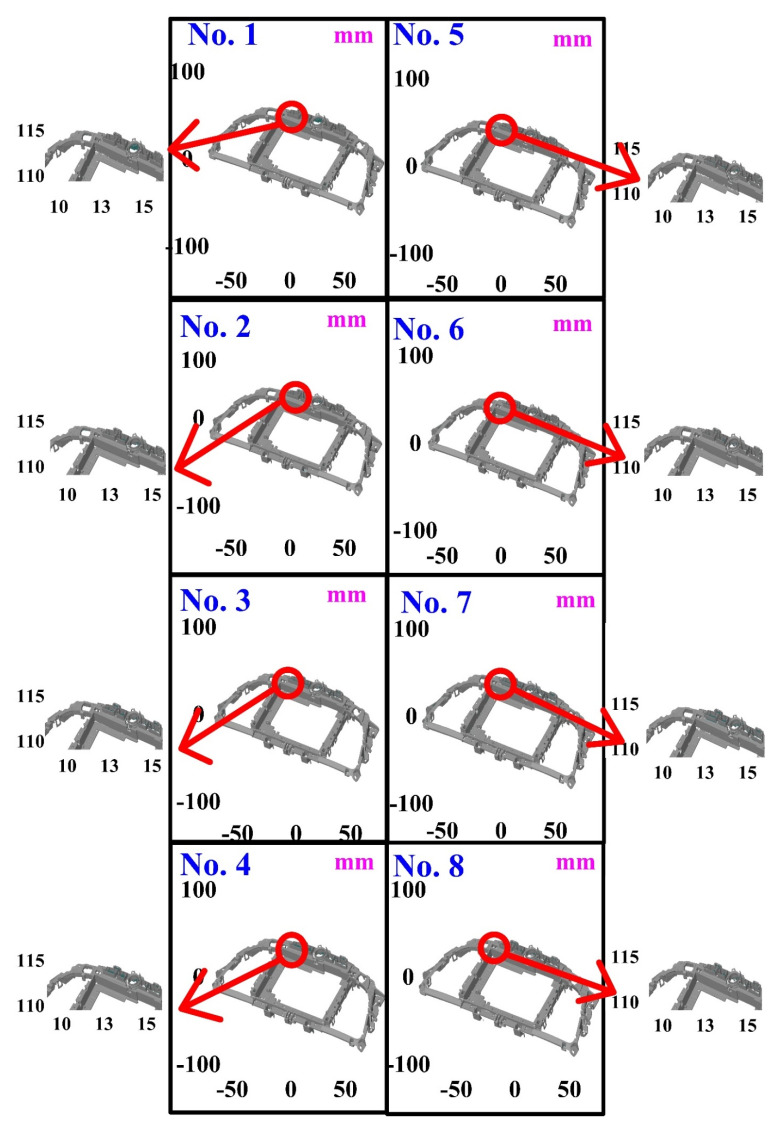
Measurement results of dark plastic part without sprayed mixture of TiO_2_ powder and ethanol.

**Figure 11:**
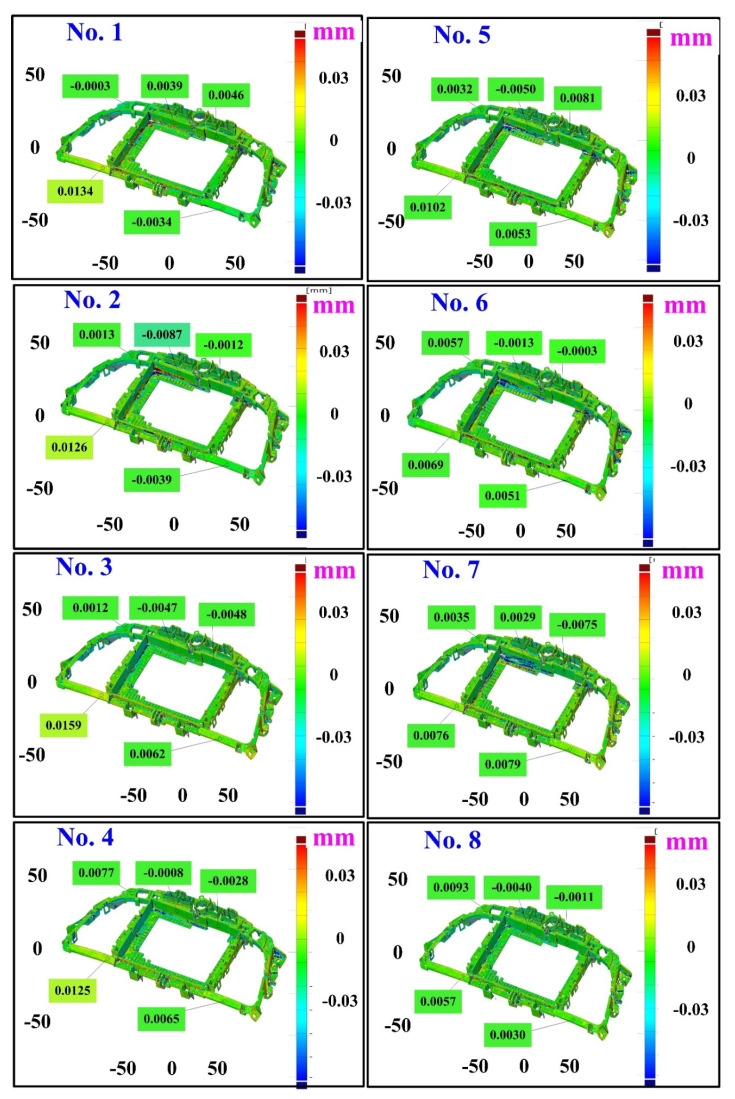
CAV results of dark plastic part. Data of five measurements in figure are only representative analysis results.

**Figure 12:**
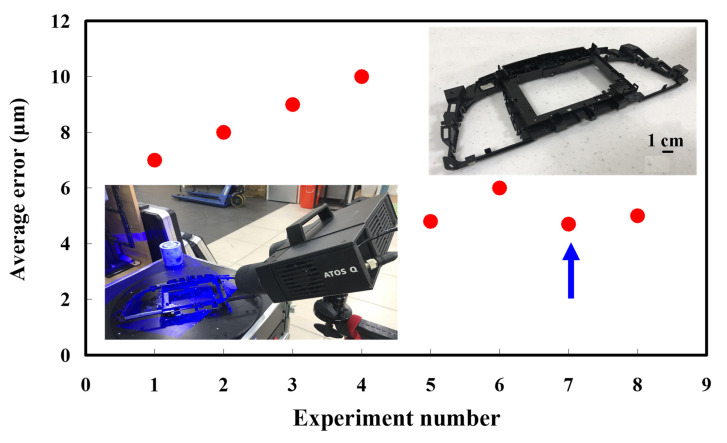
Average error of eight different measurement methods compared with conventional method for dark plastic part.

The second measurement object is a dashboard frame, which is a dark plastic part. The material of this part is made of ABS [22,23]. The characteristic of this plastic part is that the surface of the corner of the object is relatively smooth, which means that it easily produces reflections. Figure 9 shows the measurement results of the dark plastic part sprayed with a mixture of TiO_2_ powder and ethanol. The number of measurement points using high quality functions is about 1,029,655. Figure 10 shows the measurement results of the dark plastic part that was not sprayed with a mixture of TiO_2_ powder and ethanol. The number of measurement points for eight different measurement methods is approximately 900,245, 933,427, 987,288, 985,045, 920,745, 926,509, 972,174, and 963,404, respectively. The control group is considered as the nominal model and the color deviation map stands for the measurement results, which deviated from the nominal model. Figure 11 shows the CAV results of the dark plastic part. It should be noted that the data of the five measurements in the figure are only representative analysis results. Figure 12 shows the average error of the eight different measurement methods compared with conventional methods for the dark plastic part. The average size errors of the eight different measurement methods are approximately 7 µm, 8 µm, 9 µm, 10 µm, 4.8 µm, 6 µm, 4.7 µm, and 5 µm, respectively, compared with the overall measurement results of the control group. The results show that the average error of the 3D optical inspection parameters is smaller in experiments 5 to 8. It was found that the reflection of dark plastic parts is related to the ambient light because the fluorescent lamps are turned off in these experimental methods. It should be noted that the average size error of the 3D optical measurement parameters using experiment 7 is the smallest. The average size error is only 4.7 µm. Thus, the optimal measurement parameters for the dark plastic part sprayed with a mixture of TiO_2_ powder and ethanol are suggested to be full resolution, more point, reflection detection on, ambient light off, and one exposure time.

Table 3 shows an empirical technical database for 3D optical measurements of the object that was not sprayed with a mixture of TiO_2_ powder and ethanol. According to the results described above, the findings of this study can provide the greatest application potential in the plastic industry because precise 3D optical measurements of the object that was not sprayed with a mixture of TiO_2_ powder and ethanol are feasible. The time for performing 3D optical measurements is shortened greatly compared with the conventional trial and error method. In addition, the total cost of the 3D optical measurements will be significantly reduced compared with the 3D optical measurements performed under the conditions of measurement objects being sprayed with a mixture of TiO_2_ powder and ethanol. The remarkable research results in this study meet the requirements of the SDGs [24,25,26].

## 4. Conclusions

Plastic products are durable and lightweight, and can meet any requirements from customers. The non-contact measuring system is effective for measuring parts with simple or sophisticated geometries. The aim of this work was to propose an innovative, green 3D CAV technique using an ATOS optical 3D scanner. This technique can perform precise 3D optical measurements of objects that have not been sprayed with a mixture of TiO_2_ powder and ethanol. Based on the results obtained in this study, the following conclusions can be drawn:

1. The remarkable findings in this study are very practical and provide the greatest application potential in the plastic industry because precise 3D optical measurements of objects that have not been sprayed with anything has been proven to work;
2. The research results meet the requirements of the SDGs because an empirical technical database for precise 3D optical measurements of objects that have not been sprayed on their surfaces has been created;
3. An empirical technical database for two plastic parts with different surfaces has been built;
4. The dimensional average error of the two different kinds of plastic can be controlled by approximately 3 µm and 4.7 µm.

## Figures and Tables

**Table 1:** Number of test groups and measurement strategy.

No.	Resolution	Data Quality	Scan Area	Ambient Light	Exposure Time
1	Full resolution	More point	Scan all	ON	One
2	Full resolution	More point	Scan all	ON	Two
3	Full resolution	More point	Reflection detection	ON	One
4	Full resolution	More point	Reflection detection	ON	Two
5	Full resolution	More point	Scan all	OFF	One
6	Full resolution	More point	Scan all	OFF	Two
7	Full resolution	More point	Reflection detection	OFF	One
8	Full resolution	More point	Reflection detection	OFF	Two

**Table 2:** Number of measurement objects and its characteristics.

No.	Measurement Objects	Characteristics of Measurement Objects
1	Light-colored plastic part	Light-colored plastic part has reflections but the brightness and area are less than those of dark-colored plastic parts
2	Dark plastic part	Dark plastic part has reflections but the brightness and area are more than light-colored plastic parts

**Table 3:** An empirical technical database for 3D optical measurement of the object without spraying a mixture of TiO_2_ powder and ethanol.

Measurement Objects	Method	Optical Measurement Parameters	Average Size Error (µm)
Light-colored plastic part	4	Full resolution, more point, reflection detection on, ambient light on, and two exposure times	3
Dark plastic part	7	Full resolution, more point, reflection detection on, ambient light off, and one exposure time	4.7

## Data Availability

Not applicable.
